# Associations of multilingualism and language proficiency with cognitive functioning: epidemiological evidence from the SwissDEM study in community dwelling older adults and long-term care residents

**DOI:** 10.1186/s12877-023-04311-4

**Published:** 2023-10-06

**Authors:** Deborah Pacifico, Serena Sabatini, Maddalena Fiordelli, Anna Maria Annoni, Anja Frei, Milo Puhan, Gwendolyn Graf, Emiliano Albanese

**Affiliations:** 1 Institute of Public Health, Faculty of Biomedical Sciences, Università della Svizzera Italiana, Lugano, Switzerland; 2 School of Medicine, University of Nottingham, Nottingham, UK; 3 Department of Health Sciences and Medicine, University of Lucerne, Lucerne, Switzerland; 4 Epidemiology Biostatistics and Prevention Institute, University of Zurich, Zurich, Switzerland

**Keywords:** Multilingualism, Proficiency, Cognitive reserve, Cognition, Memory, Verbal fluency

## Abstract

**Background:**

We explored whether number of languages spoken and language proficiency are associated with cognitive performance among older adults living in the community and in long-term care (LTC) in Switzerland.

**Methods:**

Among study participants, 664 lived in the community in the Canton of Zurich (Mean age = 72.97 years; *SD* = 6.08), 386 lived in the community in Ticino (Mean age = 76.24 years; *SD* = 6.66), and 176 resided in LTC in Ticino (Mean age = 87.61 years; *SD* = 6.45). We recorded sociodemographic variables, number of languages spoken, language proficiency, and assessed overall cognitive performance, immediate and delayed memory, and verbal fluency with standardized tests. We used adjusted regression models.

**Results:**

A higher number of spoken languages was positively associated with overall cognitive performance, verbal fluency and immediate and delayed memory performance in community-dwelling older adults in the Cantons of Ticino and Zurich, (all p values ≤ 0.012;), but not in in older adults living in LTC homes (all p values ≥ 0.35). Higher language proficiency was associated with better memory performance among individuals living in the community in Ticino (p value = 0.003), and to better performance in verbal fluency and memory tasks in Zurich (p values *≤ 0*.002). Among LTC residents, proficiency levels were not associated with cognitive performance.

**Conclusions:**

Multilingualism and greater language proficiency were associated with better cognitive functioning in community-dwelling but not in institutionalized older adults. Multilingualism may contribute to cognitive reserve, as well as protect and delay cognitive decline in late life.

## Background

Aging is associated with changes in brain structures [1]. Some of these changes may lead to age-associated (i.e., expected) cognitive decline [2] in different cognitive domains including reasoning, memory, and attention [2, 3]. Substantial decline in one or more cognitive abilities instead may cause significant impairment in functional abilities, and autonomy [4, 5].

Cognitive and brain reserve may ward off the effects of brain changes on cognitive functions thanks to strong and extensive neuronal networks that build up across the life course [6–8]. Cognitive reserve is the result of people’s engagement in cognitively stimulating activities throughout their lives including education, intellectually demanding jobs, and complex leisure activities [9–14]. Another possible contributor to cognitive reserve is multilingualism (i.e. speaking more than one language), which is related to more efficient use of brain resources in the face of neuropathology [15, 16]. However, evidence linking multilingualism to cognitive performance is inconsistent [17, 18]. While some studies reported positive associations [8, 19–25], others found null associations [26, 27] between greater number of spoken languages and cognitive performance in older people.

Discrepancies between studies may be ascribed to the heterogeneity of study designs and measures used. Some studies explored cognitive differences between those who speak one language and those who speak two or more languages [28], other studies looked at the incremental benefit on cognition of each additional language spoken [22–24]. Several studies have found that multilingualism may delay the onset of dementia only with three or more languages spoken [20, 26, 29], which is more common for people living in multilingual countries such as South Africa, Nigeria, Kenya, Canada, Belgium, and Switzerland [20, 30].

The limited evidence on the association between language proficiency (i.e. degree of fluency) and cognitive functioning suggests that potential beneficial effects on cognitive performance occur only above a certain threshold of language proficiency [31]. Next, multilingualism may contribute to better verbal intelligence and processing speed, but benefits may not extend to other cognitive domains [24, 32]. Finally, multilingualism may be beneficial for cognition both in cognitively healthy, community-dwelling, and in cognitively impaired, institutionalized older adults [15, 16, 20, 28]. However, evidence from studies that focused on both populations concomitantly do not exist.

In Switzerland, bi- and multilingualism are common, with over 40% of people over the age of 15 regularly using more than one language [33]. Thus, Switzerland is an ideal setting to conduct research to study the association between multilingualism and cognitive abilities. We aimed to explore the association of number of languages spoken and language proficiency with overall cognitive performance, memory, and verbal fluency. We conducted our study in three distinct subsamples: people living in the community in Ticino, living in the community in the Canton of Zurich, and living in long-term care (LTC) in Ticino. These three populations vary across a number of factors including multilingualism, language proficiency, age, and cognitive function levels (lowest in LTC because cognitive impairment is a main predictor of institutionalization). We hypothesized that both a greater number of languages spoken, and high language proficiency are related to better overall cognitive performance, verbal fluency, and immediate and delayed memory in all three subsamples.

## Methods

### Study design and recruitment

We conducted a population-based cross-sectional study applying standardized methods and procedures across three study sites. The study population included Swiss individuals aged 65 years or above, both community dwelling and institutionalized in LTC facilities. We used three different recruitment strategies. First, we recruited a convenience sample of older adults living in LTC homes in Ticino. This recruitment strategy was nested within an ongoing longitudinal study in LTC homes in Ticino, COV-RISK. Residents received an informative letter about the study and were invited to register with the responsible local physician. Second, in the SwissDEM study [34] we recruited a randomized sample of people aged ≥ 65 years living in the Canton of Ticino. We sent an informative letter to older adults randomly selected from local registries, and, two weeks later, we sent an official invitation letter with instructions on how to participate in the study. Third, we leveraged the ongoing Corona Immunitas study in Zurich [35], in which randomly selected individuals from the general population were invited via letter to take part in a serosurvey. Individuals aged ≥ 65 years who agreed to participate in Corona Immunitas were asked to participate in the present study and to undergo a structured neuropsychological assessment.

We assessed participants from all three studies in face-to-face interviews between May 2021 and April 2022. Depending on participants’ preference, assessments took place at their home, in dedicated areas in local older adults’ associations, in labs at the University of Zurich and at the Università della Svizzera italiana, or in nursing homes. We used REDCap (i.e. Research Electronic Data Capture) [36, 37] on dedicated tablets with data encryption for both online and offline data collection and management.

### Ethics approval and consent to participate

This study was conducted according to the principles expressed in the Declaration of Helsinki. All participants gave informed consent to participate in the study. For participants who were physically or cognitively impaired, a legal representative signed the informed consent form. Corona Immunitas Zurich (2020 − 01247), SwissDEM (2017–02181), and COV-RISK (2020 − 01572) were authorized by local Swiss Ethics Committees. All methods were carried out in accordance with relevant guidelines and regulations.

### Data assessment

We used structured questionnaires to enquire about health and socio-demographic characteristics, including age, sex, educational level, and marital status, and previously validated neuropsychological and mental health assessments for overall cognitive performance, immediate and delayed memory, verbal fluency, and depressive symptoms [38].

#### Number of languages spoken

We assessed the self-reported number of languages spoken, and we coded answers across five categories for those living in the community (i.e. one, two, three, four, and five or more languages). Because very few participants in LTC homes reported to speak more than three languages we combined categories three, four, and five (i.e. one language, two languages, and three or more languages).

#### Self-reported language proficiency

We used one item from the Language Experience and Proficiency Questionnaire (LEAP-Q) to explore participants’ language proficiency: for each language spoken, participants rated their proficiency in speaking, understanding, writing, and reading on a Likert scale from 1 to 10. The selected item was: “On a scale from 0 to 10, please indicate your level of proficiency in speaking, understanding, and reading” [39]. We computed an indicator of self-reported language proficiency summing up the proficiency scores of each language spoken, excluding participant’s mother tongue; a similar approach has been used in the literature to investigate language proficiency in multilingual individuals [31].

#### Overall cognitive performance

To assess cognitive performance we used the Community Screening Instrument for Dementia (CSI’D’), participant part [40]. The CSI’D’ is a widely used and culturally unbiased cognitive battery conceived for population-based samples. It assesses several cognitive domains comprising orientation, memory, language expression, comprehension, and spatial constructional praxis. The total score is calculated by summing its 35 items. Higher total score (possible range: 0–35) indicates better cognitive performance.

#### Immediate and delayed memory performance

We used the CERAD ten-word list learning task to assess memory. The test consists of a list-learning paradigm in which participants listen to the interviewer who reads clearly and at slow pace a list of 10 words (one second per word). Participants are asked to recall as many words as possible; the process is repeated three times (immediate recall), and one more time five minutes later (deferred recall). The total score for both immediate (possible range: 0–30) and deferred recall (possible range: 0–10) consists in the sum of correctly remembered words. The total CERAD ten-word list learning task score is obtained from the sum of immediate and deferred recall scores (possible range: 0–40). Higher scores mirror better memory performance [41].

#### Verbal fluency

We used the CERAD animal naming test to assess semantic fluency, whereby participants have to name all the animals that come to their mind in one minute [41]. The total CERAD verbal fluency task score consisted in the number of mentioned animals. Higher scores indicate better verbal fluency.

#### Sociodemographic information

Sociodemographic variables comprised age, sex, educational level, and marital status. Educational level comprised four categories: No education/Not completed primary school/Primary school; Secondary school; High school; University certificate. Marital status comprised six categories: Single; Married; Divorced; Separated; Widowed; Civil union.

### Data analysis

We reported descriptive statistics of study variables for the three subsamples of participants living in LTCs in Ticino, in the community in Ticino, and living in the community in the Canton of Zurich. We used ANOVA and Chi squared tests for comparisons across the three different recruitment sites.

In a set of linear regression models, we quantified the association of self-reported number of languages spoken and/or self-reported language proficiency (modelled as independent variables) with overall cognitive performance, verbal fluency, and immediate and delayed memory (dependent variables). We ran unadjusted, and minimally adjusted models adding to the model the covariates age and sex. In a sensitivity analysis, self-reported language proficiency was dichotomized into low proficiency and high proficiency using the median of the whole sample’s proficiency score distribution. Linear regression models have been performed in the overall sample and separately per recruitment site (participants living in LTCs in Ticino, in the community in Ticino, and in the community in the Canton of Zurich). We also calculated standardized regression coefficients (ß, effects sizes) to quantify the magnitude of the associations and allow comparisons across cognitive outcomes; coefficients ≤ 0.09 were considered negligible, between 0.10 and 0.29 were considered small, between 0.30 and 0.49 moderate, and ≥ 0.50 large [42]. We conducted all analyses in SPSS version 26 [43] .

## Results

### Descriptive statistics

The main socio-demographic and linguistic characteristics are presented separately for the three study samples, and in Table 1.

**Table 1:** Descriptive characteristics for the three subsamples (overall sample N = 1226)

	*Living in LTC homes in Ticino* (n = 176)	*Living in the community in Ticino* (n = 386)	*Living in the community in Zurich* (n = 664)	p-value
Age, *M* (*SD*)	87.61 (6.45)	76.24 (6.65)	72.97 (6.08)	< 0.001
Women, n (%)	143 (81.3)	193 (50.0)	315 (47.4)	< 0.001
Educational level, n (%)				< 0.001
No education/ Not finished elementary school/ Primary school	42 (24.9)	34 (8.9)	12 (1.8)	
Secondary school	53 (31.4)	81 (21.1)	288 (43.3)	
High school	65 (36.9)	209 (54.4)	118 (17.8)	
University/Professional certificate	9 (5.1)	60 (15.6)	246 (37.0)	
Marital status, n (%)				< 0.001
Single	21 (11.9)	30 (7.8)	56 (8.4)	
Married	31 (17.6)	235 (61.4)	423 (63.7)	
Civil union	0 (0.0)	5 (1.3)	0 (0.0)	
Divorced/Separated	16 (9.1)	41 (10.7)	104 (15.7)	
Widowed	106 (60.2)	72 (18.8)	81 (12.2)	
Languages spoken, n (%)				< 0.001
One	66 (37.5)	46 (11.9)	54 (8.2)	
Two	61 (34.7)	88 (22.9)	98 (14.8)	
Three	33 (18.8)	115 (29.9)	188 (28.4)	
Four	13 (7.4)	100 (26.0)	210 (31.7)	
Five	3 (1.7)	34 (8.8)	89 (13.4)	
Six or more	0 (0.0)	2 (0.6)	23 (3.5)	
Language proficiency *M* (*SD*)	11.65 (7.06)	14.70 (7.83)	13.79 (7.25)	< 0.001
Overall cognitive score, *M* (*SD*)	21.40 (8.86)	31.27 (3.63)	33.80 (1.34)	< 0.001
Verbal Fluency, *M* (*SD*)	7.35 (4.71)	18.90 (6.30)	23.98 (5.94)	< 0.001
Immediate and delayed memory performance, *M* (*SD*)	8.09 (5.35)	20.98 (6.61)	28.10 (5.70)	< 0.001

LTC = Long term care

Participants living in LTC (n = 176) had a mean age of 87.61 years (SD = 6.45 years); 81.3% were women. A quarter of participants had no education, did not finish elementary school, or finished primary school only (24.9%). About one third had a secondary school (31.4%), or high school degree (38.5%), and only 5.3% obtained a University degree or professional certificate. 17.6% were married, and the majority of them were widowed (60.2%). In this group, 37.5% spoke only one language, 34.7% spoke two languages, and 27.9% spoke three or more languages.

Participants living in the community in Ticino (n = 386) had a mean age of 76.24 years (SD = 6.66 years); half were women (50%). The majority of participants in this group had at least a high school degree (70%) and were married (61.4%). Number of spoken languages varied among participants in this group: 11.9% spoke only one language, 22.9% spoke two languages, 29.9% spoke three languages, 26% spoke four languages, and 9.4% spoke five or more languages.

Participants living in the community in the Canton Zurich (n = 664) had mean age of 72.97 years (SD = 6.08 years); almost half were women (47.4%). Most participants in this group had at least a high school degree (54.8%) and were married (63.7%). Number of spoken languages varied: few (8.2%) spoke only one language, 14.8% spoke two languages, 28.4% spoke three languages, nearly a third (31.7%) spoke four languages, and 16.9% spoke five or more languages.

ANOVA and Chi squared tests showed that participants in the three recruitment sites differed for all the considered sociodemographic variables (all p-values < 0.001). Participants living in nursing homes were older, less educated, and performed poorly on cognitive task.

### Number of languages spoken and cognitive performance

The number of languages spoken was positively, and significantly associated with overall cognitive performance, immediate and delayed memory, and verbal fluency, in both unadjusted an adjusted models (all p values < 0.001). Because associations significantly differed by study site (all p values < 0.001), we proceeded with a stratified analysis (i.e. by site).

The association of multilingualism with overall cognitive performance, immediate and delayed memory, or verbal fluency was not statistically significant in older adults living in LTC homes (all p values ≥ 0.346). Among those living in the community in Ticino, in adjusted regression models, overall cognitive performance, verbal fluency and immediate and delayed memory performance were positively and significantly associated with a higher number of spoken languages (all p values *≤* 0.012). Effects were of small magnitude (ß ranging from 0.12 to 0.23). We found similar results among participants living in the community in the Canton of Zurich (all p values ≤ 0.001), and effects were of small magnitude (ß ranging from 0.14 to 0.21) (Table 2).

**Table 2:** Association of languages spoken with overall cognitive function, verbal fluency, and memory overall, in people living in the community in Zurich and Ticino, and in long-term care homes in Ticino – the SwissDEM study

	*Overall*		*Living in LTC homes in Ticino*		*Living in the community in Ticino*		*Living in the community in Zurich*	
	***B*** **(95% CI); p-value**	**ß**	***B*** **(95% CI); p-value**	**ß**	***B*** **(95% CI); p-value**	**ß**	***B*** **(95% CI); p-value**	**ß**
**Overall cognitive performance**								
**Unadjusted model**								
Languages spoken	1.45 (1.22; 1.69); <0.001	0.34	0.37 (-0.65; 1.39); 0.474	0.05	0.57 (0.26; 0.87); <0.001	0.18	0.97 (0.63; 1.31); <0.001	0.21
F	(1,1221) = 161.79; <0.001		(1,174) = 0.514; 0.474		(1,383) = 13.485; <0.001		(1,660) = 14.729; <0.001	
Adjusted R^2^	0.116		-0.003		0.031		0.020	
**Adjusted model**								
Languages spoken	0.37 (0.20; 0.54); <0.001	0.09	0.41 (-0.59; 1.412); 0.416	0.06	0.46 (0.17; 0.74); 0.002	0.15	0.15 (0.07; 0.23); <0.001	0.14
Age	-0.18 (-0.21; -0.15); <0.001	-0.26	-0.22 (-0.38; -0.07); 0.006	-0.21	-0.19 (-0.24; -0.14); <0.001	-0.34	-0.06 (-0.07; -0.04); <0.001	-0.25
Gender	-0.19 (-0.60; 0.22); 0.35	-0.02	0.62 (-1.97; 3.22); 0.637	0.04	0.17 (-0.51; 0.84); 0.626	0.02	0.09 (-0.10; 0.29); 0.350	-0.04
Sample	3.94 (3.59; 4.29); <0.001	0.53						
F	(4, 1218) = 398.74; <0.001		(3, 172) = 2.795; 0.042		(3,381) = 22.437; <0.001		(3,658) = 21.121; <0.001	
Adjusted R^2^	0.566		0.030		0.143		0.082	
**Verbal fluency**								
**Unadjusted model**								
Languages spoken	2.78 (1.94; 2.61); <0.001	0.36	0.20 (-0.49; 0.90); 0.565	0.04	0.83 (0.30; 1.40); 0.002	0.16	0.84 (0.48; 1.20); <0.001	0.18
F	(1,1221) = 178.550; <0.001		(1, 174) = 0.333; 0.565		(1, 383) = 9.418; 0.002		(1,660) = 21.121; <0.001	
Adjusted R^2^	0.127		-0.004		0.021		0.030	
**Adjusted model**								
Languages spoken	0.77 (0.50; 1.04); <0.001	0.12	0.24 (-0.45; 0.93); 0.494	0.05	0.64 (0.14; 1.14); 0.012	0.12	0.81 (0.46; 1.16); <0.001	0.17
Age	-0.28 (-0.33; -0.24); <0.001	-0.28	-0.13 (-0.24; -0.02); 0.020	-0.18	-0.32 (-0.40; -0.23); <0.001	-0.33	-0.24 (-0.31; -0.16); <0.001	-0.24
Gender	-0.17 (-0.83; 0.48); 0.600	-0.01	-0.20 (-1.99; 1.549); 0.828	− 0.02	-0.93 (-2.11; 0.24): 0.119	-0.07	0.64 (-0.23; 1.51); 0.151	0.05
Sample	5.25 (4.69; 5.81); <0.001	-0.47						
F	(4, 1218) = 328.983; <0.001		(3, 172) = 2.012; 0.114		(3, 381) = 20.864; <0.001		(3,658) = 22.454; <0.001	
Adjusted R^2^	0.518		0.017		0.134		0.089	
**Immediate and delayed memory**								
**Unadjusted model**								
Languages spoken	2.88 (2.51; 3.24); <0.001	0.41	0.34 (-0.45; 1.14); 0.396	0.06	1.52 (0.98; 2.06); <0.001	0.27	0.97 (0.63; 1.31); <0.001	0.21
F	(1,1221) = 240.304; <0.001		(1,174) = 0.724; 0.396		(1,383) = 30.253; <0.001		(1,660) = 30.888; <0.001	
Adjusted R^2^	0.164		-0.002		0.071		0.043	
**Adjusted model**								
Languages spoken	1.06 (0.80; 1.32) < 0.001	0.15	0.37 (-0.41; 1.16); 0.346	0.07	1.31 (0.80; 1.82); <0.001	0.23	0.94 (0.63; 1.31); <0.001	0.21
Age	-0.30 (-0.35; -0.26) < 0.001	-0.27	-0.18 (-0.30; -0.05); 0.005	-0.21	-0.34 (-0.43; -0.25); <0.001	-0.34	-0.24 (-0.31; -0.18); <0.001	-0.26
Gender	1.84 (1.20; 2.47) < 0.001	0.1	0.63 (-1.40; 2.65); 0.542	-0.05	0.21 (0.02; 2.41); 0.05	0.09	-0.03 (-0.30; 0.48); 0.851	-0.24
Sample	-6.98 (6.44; 7.53) < 0.001	0.56						
F	(4, 1218) = 524.575; <0.001		(3, 172) = 2.933; 0.035		(3,381) = 31.163; <0.001		(3,658) = 46.442; <0.001	
Adjusted R^2^	0.632		0.032		0.191		0.171	

Adjusted for age and sex. Note: ß = Standardized regression coefficient. SE, standard error; 95% CI, confidence intervals with lower and upper limits. LTC = Long term care. Gender 1 = male, 2 = female; sample 1 = LTC homes, 2 = people living in the community in Ticino and 3 = people living in the community in Zurich

### Language proficiency and cognitive performance

Linear regression models showed that higher language proficiency was associated with better cognitive functioning, verbal fluency, and memory performance (all p values *≤* 0.014), with positive effect modifications by study site (all p values < 0.001). In older adults living in LTC in Ticino, in the adjusted model, cognitive variables were not significantly associated with higher language proficiency (all p values ≥ 0.487) (Table 3). Among those living in the community in Ticino, memory performance was significantly and positively associated with higher language proficiency (ß*= 0.16;* p value = 0.003), but higher language proficiency was not significantly associated with overall cognitive performance and verbal fluency (p values ≥ 0.581). In the Zurich sample, language proficiency was a significant predictor of overall cognitive performance, verbal fluency and memory performance (p values ≤ 0.011). Significant associations were of small magnitude (ß ranging from 0.10 to 0.13) (Table 3).

**Table 3:** Association of number of self-reported language proficiency with overall cognitive function, verbal fluency, and memory overall, in people living in the community in Zurich and Ticino, and in long-term care homes in Ticino – the SwissDEM study

	*Overall*		*Living in LTC homes in Ticino*		*Living in the community in Ticino*		*Living in the community in Zurich*	
	***B*** **(95% CI); p-value**	**ß**	***B*** **(95% CI); p-value**	**ß**	***B*** **(95% CI); p-value**	**ß**	***B*** **(95% CI); p-value**	**ß**
**Overall cognitive performance**								
**Unadjusted model**								
Language proficiency	0.05 (0.02; 0.09); 0.006	0.09	0.05 (-0.13; 0.22); 0.589	0.05	0.00 (-0.04; 0.05); 0.901	0.01	0.02 (-0.00; 0.03); 0.012	0.10
F	(1,1018) = 7.66; 0.006		(1, 102) = 0.293; 0.589		(1, 310) = 0.015; 0.901		(1; 602) = 6.411; 0.012	
Adjusted R^2^	0.006		-0.007		-0.003		0.009	
**Adjusted model**								
Language proficiency	0.03 (0.01; 0.06) 0.014	0.05	0.04 (-0.13; 0.21); 0.629	0.01	0.00 (-0.04; 0.05); 0.857	0.010	0.02 (-0.00; 0.03); 0.011	0.10
Age	-0.16 (-0.19; -0.13) < 0.001	-0.27	-0.24 (-0.42; -0.05); 0.011	-0.25	-0.16 (-0.22; -0.11); <0.001	− 0.33	-0.05 (-0.06; 0.03); <0.001	-0.23
Gender	-0.49 (-0.87; -0.10) = 0.013	-0.05	-2.16 (-5.70; 1.38); 0.857	-0.12	-0.05 (-0.74; 0.65); 0.889	− 0.01	-0.07 (-0.13; 0.27); 0.507	-0.03
Sample	3.68 (3.35; 4.01) < 0.001	0.55						
F	(4, 1015) = 310.429; <0.001		(3, 100) = 3.066; 0.031		(3, 308) = 12.618; <0.001		(3, 600) = 13.666; <0.001	
Adjusted R^2^	0.548		0.057		0.101		0.058	
**Verbal fluency**								
**Unadjusted model**								
Language proficiency	0.11 (0.05; 0.18); <0.001	0.11	0.01 (-0.13; 0.12); 0.903	-0.01	0.03 (-0.07; 0.12): 0.581	0.03	0.11 (0.05; 0.18); 0.001	0.13
F	(1,1018) = 12.240; <0.001	(1,1024) = 13.801; <0.001	(1, 102) = 0.015; 0.903		(1, 310) = 0.305; 0.581		(1, 602) = 11.077, 0.001	
Adjusted R^2^	0.011	0.012	0.010		-0.002		0.016	
**Adjusted model**								
Language proficiency	0.08 (0.03; 0.13) 0.001	0.08	-0.02 (-0.1; 0.10); 0.748	-0.03	0.03 (-0.06; 0.11); 0.521	0.03	0.11 (0.05; 0.17); 0.001	0.13
Age	-0.30 (-0.36; -0.25) < 0.001	− 0.30	-0.15 (-0.28; -0.02); 0.021	-0.22	-0.32 (-0.43; -0.22); <0.001	-0.33	-0.25 (-0.32; -0.17); <0.001	-0.25
Gender	-0.50 (-1.22; 0.23) 0.178	− 0.03	-3.20 (-5.69; -0.713); 0.012	-0.24	-1.49; (-2.84; -0.14): 0.030	-0.12	0.61 (-0.30, 1.53); 0.189	0.05
Sample	5.27 (4.65; 5.90) < 0.001	0.46						
F	(4, 1015) = 210.034; <0.001		(3, 100) = 4.496; 0.005		(3, 308) = 15.006; <0.001		(3, 600) = 18.654; <0.001	
Adjusted R^2^	0.451		0.092		0.119		0.081	
**Immediate and delayed memory**								
**Unadjusted model**								
Language proficiency	0.14 (0.07; 0.21); <0.001	0.12	-0.05 (-0.17; 0.14); 0.518	-0.05	0.13 (0.04; 0.22): 0.005	0.16	0.09 (0.03; 0.15); 0.003	0.12
F	(1,1018) = 15.642; <0.001		(1, 102) = 0.420; 0.518		(1, 310) = 7.852; 0.005		(1, 602) = 8.742, 0.003	
Adjusted R^2^	0.014		-0.006		0.022		0.013	
**Adjusted model**								
Language proficiency	0.10 (0.06; 0.15) < 0.001	0.09	-0.05 (-0.17; 0.11); 0.487	-0.07	0.13 (0.05; 0.22); 0.003	0.16	0.09 (0.04; 0.15); 0.002	0.12
Age	-0.31 (-0.36; -0.26) < 0.001	− 0.28	-0.22 (-0.46; -0.09); 0.003	-0.29	-0.34 (-0.44; -0.23); <0.001	-0.34	-0.25 (-0.31; -0.18); <0.001	-0.27
Gender	1.69 (0.99; 2.38) < 0.001	0.10	-1.10 (-4.28; 1.55); 0.452	-0.07	0.60; (-0.77; 1.96): 0.389	0.05	2.84 (2.01, 3.66); <0.001	0.25
Sample	7.13 (6.54; 7.73) < 0.001	0.57						
F	(4, 1015) = 335.495; <0.001		(3, 100) = 3.551; 0.017		(3, 308) = 16.592; <0.001		(3, 600) = 37.090, < 0.001	
Adjusted R^2^	0.568		0.069		0.131		0.152	

Adjusted for age and sex. Note: ß = Standardized regression coefficient. SE, standard error; 95% CI, confidence intervals with lower and upper limits. LTC = Long term care. Gender 1 = male, 2 = female; sample 1 = LTC homes, 2 = people living in the community in Ticino and 3 = people living in the community in Zurich

## Discussion

We explored the associations of multilingualism with overall cognitive function, memory, and verbal fluency in community-dwelling and institutionalized older adults in Switzerland, a multilingual European country. We found that a greater number of languages spoken, and higher language proficiency were associated with better cognitive function across different domains. Our results suggest that multilingualism may be beneficial for cognitive function in community-dwelling older adults, and this protective effect is no longer present in institutionalized older adults who were older and more likely cognitively impaired.

Our findings that among older people living in the community an increasing number of languages spoken are associated to better overall cognitive performance, better verbal fluency, and better immediate and delayed memory are in line with findings from existing studies documenting the beneficial effect of multilingualism on cognitive performance, and suggesting that multilingualism might contribute to cognitive reserve [8, 19, 21–25].

Consistent with the limited existing evidence linking greater language proficiency with better cognition [31], we found that high language proficiency was associated with better immediate and delayed memory performance and better verbal fluency among those living in the community.

There were no significant associations between multilingualism and language proficiency with cognitive functioning in older people residing in LTCs. These results are novel, and may be due to the fact that older adults living in LTCs were on average older and had lower cognitive functioning compared to the subsamples living in the community. Assuming that multilingualism contributes to cognitive reserve, our findings are consistent with previous evidence on the lack of protective effects of cognitive reserve on cognition in severe cognitively impaired older adults [7]. Cognitive impairment is a main driver of institutionalization in older adults [44]. The more advanced stages of cognitive impairment in LCTs residents were likely related to a greater extent of structural and functional brain damage, which may hamper and compromise the buffering effect of cognitive reserve on cognitive impairment [7, 45].

Future studies should consider evaluating language competencies, such as dominance, age of acquisition, language immersion and might profit from objective language assessment, and may include functional neuroimaging techniques and experimental cognitive tasks to characterize brain structure and function.

Dementia prevention is key, and in community-dwelling older adults, multilingualism may contribute to and maintain cognitive reserve, which is thought to buffer the deleterious effect on cognitive function of the neuropathology [29]. Population-level preventive interventions and programs aimed at stimulating and maintaining multilingualism across the life course can contribute to delay the onset of symptoms of dementia, above and beyond the effect of education.

This study has several limitations that need to be acknowledged. First, language proficiency was self-reported. Although information bias cannot be excluded, whereby participants might have reported inaccurate information about their level, this was unlikely to be influenced by their cognitive performance. Second, to assess cognition we used the CSI’D’ participant part [40] which was designed as a screening tool for pathological cognitive decline. A ceiling effect in cognitively healthy older adults living in the community may have contributed to dilute the magnitude of the associations in this population. Nevertheless, we also included verbal fluency and memory specific tasks in our battery. Third, residual confounding is possible because we purposely did not adjust for educational level in our models, however, education is commonly used as a proxy of cognitive reserve.

Strengths of the study include the representativeness of the study samples, standard, previously validated cognitive assessments conducted by purposely trained interviewers, and its novelty. Indeed, this is the first study linking language proficiency with cognitive functioning in Switzerland (an official multilingual country), to include both community-dwelling and institutionalized older adults.

## Conclusion

Taken together, the findings from this study suggest that both the greater number of language spoken and higher levels of language proficiency may be protective factors of cognition in late life. Future policies and dementia preventive strategies may entail interventions that stimulate and maintain multilingualism as an independent contributor to cognitive reserve in populations.

## Data Availability

All data used will be made available to promote transparency and to allow replications upon request to the last author (emiliano.albanese@usi.ch). Data for the SwissDEM study will be available in compliance with SNSF requirements. Data from COV-RISK interviews will be made available upon reasonable request to the last author. Deidentified individual participant data underlying the findings of this study will be available for researchers submitting a methodologically sound proposal to achieve the aims of the proposal after publication of this article. Proposals should be directed at the last author.

## List of abbreviations

LTC: Long Term Care
CSI’D’: Community Screening Instrument for Dementia
